# Methodological Issues in Antifungal Susceptibility Testing of *Malassezia pachydermatis*

**DOI:** 10.3390/jof3030037

**Published:** 2017-07-05

**Authors:** Andrea Peano, Mario Pasquetti, Paolo Tizzani, Elisa Chiavassa, Jacques Guillot, Elizabeth Johnson

**Affiliations:** 1 Dipartimento diScienze Veterinarie, Università di Torino, Largo Paolo Braccini 2, 10095 Grugliasco, Italy; mario.pasquetti@unito.it (M.P.); paolo.tizzani@unito.it (P.T.); elisac_85@hotmail.it (E.C.); 2 Ecole nationale Vétérinaire d’Alfort, Dynamyc Research Group, EnvA, Université, Maisons-Alfort 94700, France; jacques.guillot@vet-alfort.fr; 3 Public Health England Mycology Reference Laboratory, Kingsdown, Bristol BS2 8EL, UK; Elizabeth.Johnson@uhbristol.nhs.uk

**Keywords:** *Malassezia pachydermatis*, susceptibility testing, broth microdilution, disk diffusion, minimum inhibitory concentration (MIC)

## Abstract

Reference methods for antifungal susceptibility testing of yeasts have been developed by the Clinical and Laboratory Standards Institute (CLSI) and the European Committee on Antibiotic Susceptibility Testing (EUCAST). These methods are intended to test the main pathogenic yeasts that cause invasive infections, namely *Candida* spp. and *Cryptococcus* *neoformans*, while testing other yeast species introduces several additional problems in standardization not addressed by these reference procedures. As a consequence, a number of procedures have been employed in the literature to test the antifungal susceptibility of *Malassezia pachydermatis*. This has resulted in conflicting results. The aim of the present study is to review the procedures and the technical parameters (growth media, inoculum preparation, temperature and length of incubation, method of reading) employed for susceptibility testing of *M. pachydermatis*, and when possible, to propose recommendations for or against their use. Such information may be useful for the future development of a reference assay.

## 1. Introduction

The genus *Malassezia* is now known to include different species of yeast, many of which have been associated with various diseases in humans and animals [1,2]. *Malassezia pachydermatis* is the lone lipophilic, but not lipid-dependent, species of this genus. The other species show an absolute requirement for long fatty acid chains and specific procedures are required for their isolation [3].

*M. pachydermatis* colonizes the skin and mucosal sites of healthy dogs and cats. Favorable growth conditions in the local environment allow excessive multiplication of this organism, which may then function as an opportunistic secondary pathogen. *Malassezia* dermatitis and otitis, inflammatory diseases associated with elevated populations of *M. pachydermatis* on the skin and in the ear canal of dogs and cats, have been recognized with increasing frequency [4,5]. The underlying conditions leading to the yeast overgrowth include hypersensitivity diseases (atopy, adverse cutaneous food reactions, flea bite hypersensitivity, and contact allergy), cornification disorders, ectoparasite infection, bacterial pyoderma, and endocrine diseases (hyperadrenocorticism, hypothyroidism, diabetes mellitus). Moreover, a hypersensitivity response to the yeast itself is likely to occur in many allergic dogs [4,5,6]. Current treatment options for *Malassezia* dermatitis/otitis in dogs include systemic and/or topical therapy with a number of antifungal agents, in addition to various antiseptics. Azole derivatives (itraconazole—ITZ; ketoconazole—KTZ; miconazole—MCZ; clotrimazole—CTZ etc.) are the more common choice, though other agents belonging to various chemical classes are also used (terbinafine—TER; thiabendazole—TBZ) [4,5,7,8,9]. Although the dog is the main host, *M. pachydermatis* may be found to cause bloodstream infections in humans. Infections have been linked to the administration of lipids through intravenous catheters, especially in infants in intensive care units. *M. pachydermatis* is considered to be transferred from a household pet as it is rarely isolated from normal human skin, but may be transferred on the hands of healthcare workers or family members [2,10,11,12,13,14]. Fungemia by *M. pachydermatis* in human patients is treated with azoles (e.g., fluconazole—FCZ) and other agents (e.g., amphotericin B—AMB) [2,11].

Based on the results of in vitro susceptibility tests, some studies have claimed resistance towards various agents for variable proportions of strains of *M. pachydermatis* isolated from dogs (e.g., 8% [15,16] and 20% [17] towards ITZ; 14% towards TBZ [18]; 24% towards KTZ [19]; 4% towards CTZ, MCZ, and nystatin (NYS) [20]). However, this issue remains controversial for now, principally due to the lack of a specific reference procedure for antifungal susceptibility testing (AFST). Therefore, a strain considered resistant using a certain method may appear susceptible (and vice-versa) under different test conditions.

This review aims to describe and discuss past experiences concerned with AFST of *M. pachydermatis*, with the intention of identifying the technical parameters that might be more suitable in order to increase the end-point evaluation and reproducibility of susceptibility tests for this organism. Such information may represent a good starting point for the future development of a reference assay.

## 2. Current Status of Antifungal Susceptibility Testing Methods

Until recently, the techniques for antifungal susceptibility testing were not standardized, consequently inter- and even intra-laboratory reproducibility was poor. Different testing variables, including test format, inoculum size, test medium composition, temperature, duration of incubation, and endpoint determination, are known to have an impact on in vitro determinations [21,22,23,24]. With regard to yeasts, the first properly optimized and standardized method was a broth macrodilution method developed by the Clinical and Laboratory Standards Institute (CLSI) (formerly the National Committee for Clinical Laboratory Standards). This proved unwieldy for testing large numbers of isolates, and it was later adapted to allow for a microdilution format in microtitre plates, and the updated version of this method, reported in CLSI documents M27-A3 [25] and M27-S4 [26], is now a widely accepted standard [21,22,23,24,27]. The method, intended for testing *Candida* spp. and *Cryptococcus neoformans*, relies on measurements of growth inhibition during exposure over a defined time period to a range of doubling drug concentrations diluted in liquid medium, with results expressed as the minimum concentration of the drug able to inhibit fungal growth (minimum inhibitory concentration (MIC)). A standard antifungal disk diffusion susceptibility testing method for *Candida* vs. some antifungal agents is now also available [27,28,29]. The European Committee on Antibiotic Susceptibility Testing (EUCAST) has also published guidelines for testing *Candida* isolates [30,31]. The EUCAST method is principally similar to the CLSI M27-A3 assay with modifications concerning some of the test parameters [28] (Table 1).

Thanks to the development of these reference methods for AFST, it is now possible to produce in vitro susceptibility results that are comparable between laboratories and allow epidemiological analyses at the national and even international level. In addition, the utility of antifungal susceptibility tests as an adjunct in optimizing the treatment of candidiasis has now been validated, at least for some clinical presentations/drug combinations [28]. However, in terms of predicting the outcome of therapy, several factors (e.g., pharmacokinetics of drugs, the immune system of the host, virulence factors of the infecting microorganism, etc.) have been shown to outweigh the importance of antifungal susceptibility testing [24,27,32]. Accordingly, the interpretative breakpoints now available for *Candida* spp. have been based on a number of pharmacodynamic and pharmacokinetic analyses (e.g., the evaluation of the duration of the dosing interval where the drug concentration in the tissue remains above the MIC for the infecting pathogen and the ratio of the area under the time–concentration curve to the MIC), but the proof of their validity has ultimately come from analysis of the in vitro–in vivo correlation in clinical practice [24]. In this regard, the so-called “90-60 rule”, which maintains that infections due to susceptible (S) strains of *Candida* spp. respond to appropriate therapy in ~90% of cases, whereas infections due to resistant (R) strains respond in ~60% of cases, well illustrates that in vitro susceptibility does not always predict a successful therapy while in vitro resistance often, but not always, predicts therapeutic failure [22,27].

Modifications of the available methods as well as other methodologies that might have particular advantages, such as ease of performance, economy, or more rapid results are also being intensively studied [28]. For example, the agar dilution method is a conventional method that has been studied for various antifungal agent/yeast species combinations. Although still unstandardized, it has been shown to produce good correlation with microdilution methods in most of the comparative studies [28]. An agar dilution method may be of interest for some difficult-to-grow fungi and it is indeed under investigation for lipid-dependent *Malassezia* species [28]. Another example is the E-test^®^, and more recently the introduction of other commercial gradient strips, in which a plastic test strip is impregnated with a continuous concentration gradient of an antifungal agent. This way, an MIC can be obtained in an agar diffusion test, by considering where the border of the inhibition zone intercepts the graded MIC scale on the E-test strip. The agreement of E-test with the CLSI reference method is variable, but frequently above acceptable limits [27,28].

## 3. Antifungal Susceptibility Testing of *Malassezia pachydermatis*

Compared with the extensive work done for *Candida* and *Cryptococcus*, the value of in vitro susceptibility testing has been much less comprehensively investigated for *M. pachydermatis*. As a result, standard parameters and guidelines specifically dedicated to this yeast are not available yet and neither are the interpretive criteria. Moreover, the conditions employed in the CLSI/EUCAST methods are almost universally accepted to not be suitable for *M. pachydermatis*, particularly due to the lipid-free medium (RPMI broth), which does not support adequate growth of the yeast [33,34]. Other problems are the slower growth rate compared to that of *Candida* species and the tendency to form clusters [34]. Therefore, different adjustments have been adopted in the literature [15,16,17,18,19,20,33,34,35,36,37,38,39,40,41,42,43,44,45,46,47,48,49,50,51,52,53,54,55,56,57,58,59,60,61,62,63,64,65]. Unsurprisingly, this has resulted in conflicting results. For example, Table 2 reports MICs obtained for some antifungals belonging to different chemical classes and with different mechanisms of action.

This “method dependency” of MICs is clearly shown by the highly variable results obtained by different authors for a reference strain of the yeast (*M. pachydermatis* CBS 1879) (Table 2). This conflicting situation applies also to studies that employed procedures based on agar diffusion from disks. For example, thiabendazole (TBZ) was completely ineffective in a disk diffusion test [48] whilst a recent study reported MIC values of the agent for all strains tested [34].

### 3.1. The Test Conditions

Various test formats have been employed at different times. The most popular approach was the broth microdilution methodology (BMD), followed by agar dilution (AD), disk diffusion (ADiff), Etest, and broth macrodilution (BMaD) techniques. Some authors compared two different test formats. A detailed description of the technical parameters adopted thus far is provided in the online version of this article (Table S1).

#### 3.1.1. The Growth Medium in Broth-Based Techniques

**Figure 1:**
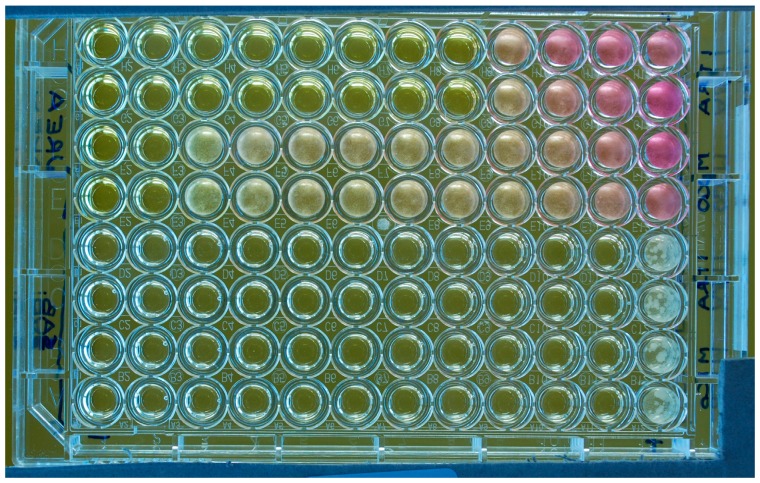
Microtitre plate (flat-bottom wells) illustrating susceptibility testing of a *M. pachydermatis* isolate against two antifungal drugs (ITZ: rows 1–2 and 5–6 and MCZ: rows 3–4 and 7–8) tested in duplicate with two different media (Urea Christensen Broth with Tween 40 and 80 as lipid source, rows 1–4; lipid-free Sabouraud broth, rows 5–8). The last column on the right comprises the drug-free growth controls. The last column on the left is the negative control (no yeast inoculum; no drug). The rows of wells contain doubling dilutions of the drugs from 4 to 0.007 µg/mL with the highest concentration to the left of the plate. For this experiment, the MICs were 0.06 (ITZ) and 4 (MCZ) µg/mL if tested in the lipid-supplemented broth; or 0.007 µg/mL for both drugs if tested in the lipid-free broth. In this latter medium, a yeast growth is visible only in the control wells. A partial color change due to the urease activity of the yeast and consequent rise of pH, is visible in some wells. It can be also noted that the yeast produced a diffuse turbidity in the wells.

Most of the growth media were formulations with lipid components included to enhance the yeast growth. Often these lipid sources (tween, glycerol, olive oil, oleic acid, ox bile, or cow’s milk fat), were added to a medium “base”, for example Christensen’s Urea, Sabouraud’s, and RPMI medium [15,34,40,56,57,58], while occasionally some authors employed media specifically developed for culturing lipid-dependent *Malassezia* species, such as Leeming-Notman and Dixon’s medium [43,54], that are very complex and contain a large quantity of lipid components. Regardless of the complexity of the medium, almost all authors who used broth-dilution procedures appear to agree that a certain lipid supplementation is mandatory when testing *M. pachydermatis* in a liquid medium. This supplementation was instead unnecessary in many studies in which solid media were employed. This agrees with the fact that although *M. pachydermatis* is distinguished within the *Malassezia* genus for its capacity to grow in vitro without supplementation with fatty acids, it generally exhibits this feature if cultured on solid media (e.g., Sabouraud Dextrose agar, SDA) [66], while in a lipid-free broth it often fails to grow or grows poorly. In such a nutritionally suboptimal situation the antifungal activity may thus be exaggerated. Such an effect, especially a partial growth, could be highly misleading when interpreting the susceptibility test results. In this situation the yeast might also grow to a certain extent in the control drug-free wells/tubes and this could suggest to an operator that a test was carried out correctly, while the activity of the drug under examination was probably overestimated (Figure 1).

Another fundamental feature expected from a medium employed for susceptibility tests is the lack of interference towards the drugs under testing. In this regard, several examples exist of obfuscation of the in vitro activity of various antifungals by some complex, undefined (and undefinable) formulations [67,68,69]. Analysis of past results seems to indicate that the growth media employed to test *M. pachydermatis*, including even the most complex ones, may not actually have a great impact on the in vitro activity of the antifungal drugs under testing. If there had been obfuscation of activity then we would have expected reports of very high MICs (or even no inhibition at all) for isolates tested in a given medium. Nonetheless, it seems logical that a medium selected for susceptibility testing (of *M. pachydermatis*, or of any organism) should first undergo a verification of the “absence of antagonism”, whereas only a few media employed for *M. pachydermatis* testing have been assessed in this way. In particular, Christensen’s broth with Tween 40/80, Sabouraud Broth with Tween 80 and RPMI with glycerol, and Tween 20 and ox bile were proven to not antagonize the drugs under testing on the basis that the MIC results for quality control strains belonging to other yeast species—*Candida krusei* ATCC 6258, *C. neoformans* ATCC 90112, and *Candida parapsilosis* ATCC 22019—were within the expected ranges obtained in RPMI by the CLSI reference method [33,57,58]. However, it is important to note that one of these media (lipid-supplemented RPMI) was shown to not produce a satisfactory yeast growth in one study [33].

The detection and characterization of antagonism should be facilitated with a chemically defined fully synthetic medium. Accordingly, CLSI/EUCAST elected to test *Candida* and *Cryptococcus* in RPMI broth, that is a fully synthetic medium, thus with a definable and reproducible formula, and is inert towards antifungal activity [28]. Unfortunately, such a medium is traditionally considered unsuitable for *Malassezia* (including *M. pachydermatis*) testing, owing to its lack of lipid supplementation. Thus, the recent reports of Jesus et al. (2011) [17] and Weiler et al. (2013) [64], who claimed to have tested *M. pachydermatis* in RPMI broth, are surprising. The explanation proposed in one of the studies [17], that the subcultures that were performed on a lipid enriched medium (Dixon agar) prior to testing avoided the depletion of lipid reserves of the yeast and allowed the subsequent growth in RPMI, is intriguing but requires further confirmation. Moreover, the authors [17] supported their findings by the observation that *M. pachydermatis* can be separated from the other *Malassezia* species by its ability to grow on lipid-free media. This is undoubtedly true, but such ability is generally only displayed on solid media, while in liquid medium some growth may occur but is unlikely to be adequately vigorous to provide suitable conditions for testing the activity of an antifungal drug. Regardless of the potential impact of the lipid supplementation on the drug’s activity, it is necessary to consider that this enrichment may have another negative implication. Lipid-enriched media are frequently turbid, which would make it difficult to assess the turbidity due to the yeast growth and therefore accurately assess the endpoint (see Section 3.1.6).

#### 3.1.2. The Growth Medium in Agar Dilution Assays

With regard to the agar dilution methodology, Gupta et al. (2000) [45] reported that Leeming–Notman agar (LNA), as a very complex, undefined medium specifically developed for culturing lipid-dependent *Malassezia* species, did not interfere with drug activity. This conclusion was drawn from the observation of similar MIC values for certain *Malassezia* species (*M. restricta*, *M. obtusa* and *M. globosa*) obtained in LNA and the defined Diagnostic Sensitivity Testing (DST; Oxoid, UK.) agar. The lack of drug antagonism by LNA was then confirmed, as noted by the authors themselves, by the low MICs of the antifungal drugs tested against most of the isolates.

#### 3.1.3. The Growth Medium in Agar Diffusion Assays

**Figure 2:**
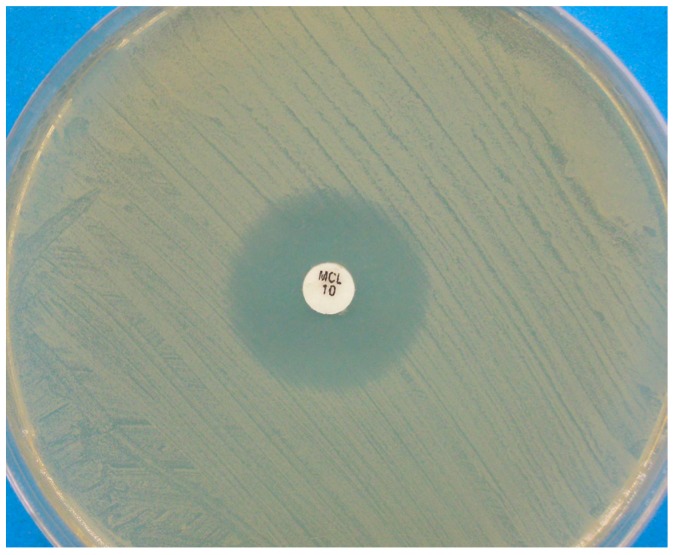
Disk diffusion assay for a *Malassezia pachydermatis* strain against miconazole, performed on Mueller-Hinton agar supplemented with 2% glucose and methylene blue. A clear inhibition zone edge is visible.

Concerning the method of agar diffusion by disks, various media were employed, i.e., Sabouraud dextrose agar (SDA), SDA with 1% Tween 80 and 1.5% yeast extract, and Casitone agar [20,36,47,48,49]. Although these media are likely to adequately support the growth of *Malassezia*, they were not evaluated with regard to their fitness in producing clear inhibition zone edges. The medium recommended in the CLSI reference assay (document M44-A2) is Mueller–Hinton (MH) agar supplemented with 2% glucose (G) and methylene blue (M). This medium allows obtaining clear inhibition zone edges and less intrazonal growth, enabling easy interpretation of the inhibition zone diameters [29]. Findings of a recent study [55] support the validity of this medium also for susceptibility testing of *M. pachydermatis* with agar diffusion procedures against two azole drugs (CTZ and MCZ), provided that the yeast inoculum is prepared with a lipid source (Tween 40 and Tween 80). With this medium, the inhibition zones were definite and clear, facilitating the measurement of zone size and minimizing subjectivity. Moreover, the inhibition zones correlated with the MIC values obtained in a broth dilution assay [55] (Figure 2).

MH agar with glucose and methylene blue might be preferable to SDA, that was employed in past studies of *M. pachydermatis* using the disk diffusion method, SDA has been reported, even though in a different test format (agar dilution assay), to obfuscate the antifungal activity of some antifungal agents, such as 5-fluorocytosine, CTZ, and MCZ [67,68].

With regards to the E-test method, the growth media employed were Yeast nitrogen agar [41], SDA [19,53], RPMI 1640 agar with ox bile, glycerol, glycerol monostearate and Tween 20 [62], Dixon agar [54], and SDA with Tween 80 [16] or Tween 40 [63]. No author reported particular problems using these media (e.g., poor growth, difficulty in the interpretation of inhibition zones, etc.).

#### 3.1.4. The Inoculum Preparation

This parameter has a recognized impact on in vitro determinations, and a detailed procedure has been developed to allow the production of definite and reproducible inocula of *Candida* spp. and *Cryptococcus* spp. [27].

For *M. pachydermatis*, most of the authors agree that, in order to counteract the slower growth rate of this organism compared to that of the *Candida* species, the inoculum size should be increased compared to that recommended in the CLSI documents (Table 1). This should provide a suitable growth of the yeast, allowing for easy interpretation of MICs/inhibition zones after 2–3 days of incubation. Instead, lower-concentration inocula have been employed on occasion (see Table S1). However, the problem centres not so much on the final inoculum size to be employed, but rather its standardization and reproducibility. There are two main reasons for this. The first is the tendency of *M. pachydermatis* cells to form clumps, owing to its butyrous nature, which makes it difficult to produce homogeneous cell suspensions, thus yielding inconsistent CFU results [70]. Second, in order to adjust the cell suspensions to the desired concentration, the density of the suspension should be assessed by a spectrophotometer. However, while parameters for this calculation are defined for *Candida* spp. and *Cryptococcus* spp., with regard to *M. pachydermatis* it is still unclear how to relate with precision of the absolute optical density (OD) value to the corresponding CFU. With regards to the first difficulty, both physical and/or chemical methods have been reported to minimize clumping. Methods that have been employed include the use of dispersing agents, such as Tween, which is principally used as a lipid source but it is also a mild detergent, and vortexing with glass beads [34,62]; using Tween alone [58]; and employing a glass homogenizer [50]. An ultrasonic homogenizer has been also reported to efficiently disperse clumps of *M. pachydermatis*, without affecting the yeast viability [70]. Issues surrounding standardization remain to be clarified. Various OD values at different wavelengths have been employed to calculate the final quantity of *M. pachydermatis* cells in suspension. For example, according to Murai et al. (2002) [50] a suspension matching a transmittance of 1.0 at 660 nm will yield 2.5 × 10^6^ cells/mL, while Rincon et al. (2006) [58] and Pietschmann et al. (2008) [56] suggested respectively that an absorbance of 0.425 to 0.435 at 530 nm wavelength and of 0.42 to 0.43 at 530 nm wavelength will yield a suspension containing 10^6^ CFU/mL. No method has been further validated by inter-laboratory comparisons to produce a consensus procedure.

Another technique has been proposed to adjust inocula, i.e., by performing quantitative viable counts, that is by culturing progressive dilutions of the original suspension and counting the number of CFU/mL thus obtained [43]. However, these viable counts, which relate the suspension density to the number of yeast cells present in it, are necessarily retrospective. They allow the inoculum concentration to be determined only after some days of incubation. Therefore, the inoculum can only be adjusted for use in tests after a prolonged incubation of the original suspension which, in the meantime, may have reduced viability.

Another issue that may be worth standardizing is the type of agar on which subcultures should be performed to obtain colonies for susceptibility tests and the number of subcultures that should be performed before testing. Repeated passages may enhance viability and subsequently lead to optimal growth in test media, however this will also delay the results of the tests. In this regard, we have already underlined the suggested use of subcultures on lipid-enriched media to allow for the subsequent growth in lipid free broths [17], that are less likely to interfere with the activity of the antifungals under testing.

In the CLSI disk diffusion assay, the recommended inoculum is equal to 0.5 McFarland standard (1 × 10^6^–5 × 10^6^ CFU/mL) [27,29] (Table 1). For this type of assay, with regard to *M. pachydermatis*, this inoculum was shown to not provide adequate confluent growth on agar plates, and higher-concentration inoculum (1 × 10^7^–5 × 10^7^ CFU/mL)—prepared with a lipid enrichment—was thus necessary [55]. Bernardo et al. (1998) [36] reported using an inoculum corresponding to a 2 McFarland turbidity standard, without specifying the corresponding number of colony forming units (CFU).

#### 3.1.5. Temperature and Length of Incubation

With a temperature between 32 and 37 °C, no more than 2 or 3 days are generally needed to obtain an adequate growth of *M. pachydermatis* in vitro. Thus, it should be possible to read MICs/inhibition zones at 72 h or even 48 h. Indeed, most studies adopted these times of reading [15,16,17,18,33,34,35,36,37,39,40,41,42,43,44,47,49,50,51,52,53,54,55,56,57,58,59,61,62,63,64,65]. However, extended incubation times, up to 7 days, were sometimes reported as necessary, especially in cases where the AD method was employed [38,45,51,60]. While any length of incubation is potentially suitable, provided that it allows an easy interpretation of MICs or inhibition zones and allows the production of reproducible results, in order to facilitate the timely production of results to enhance clinical management, shorter incubation times are preferable provided that the results are consistent and clinically relevant. In this regard, a recent study suggests that a 48 h incubation may be now recommended since no statistically significant differences were noted between 48 and 72 h MIC values [33].

#### 3.1.6. The Method of End-Point Determination in Broth- and Agar-Dilution Assays

**Figure 3:**
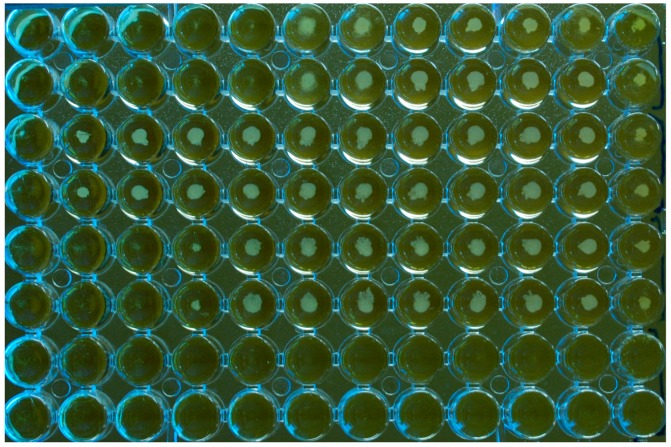
Microplate (U-bottom wells) for susceptibility testing of a *M. pachydermatis* isolate against three antifungal drugs tested in duplicate, MCZ (rows 1 and 2); TBD (rows 3 and 4); CTZ (rows 5 and 6). Growth medium: Christensen Broth with Tween 40 and 80 as a lipid source. The last column on the right comprises the drug-free growth controls. The last column on the left is the negative control (no yeast inoculum; no drug) and the two bottom rows are also inoculum free. The rows of wells contain doubling dilutions of the drugs from 16 to 0.03 µg/mL with the highest concentration to the left of the plate. For this experiment, the MICs were 2, >16, and 4 µg/mL, respectively, for MCZ, TBD, and CTZ. The plate was not agitated prior to reading. It can be seen how the yeast isolate formed button-like deposits in the wells.

For broth susceptibility testing, the turbidity of the medium, by visual assessment, is recommended as an indicator of fungal growth in the CLSI standard. As a modification, spectrophotometric reading has been studied by several investigators, resulting in favorable agreement rates with visual evaluation in general, and this method of assessment is employed in the EUCAST tests [27,28]. With regards to *M. pachydermatis*, as discussed, its tendency to form clusters makes it form a button-like deposit in the tubes and in the microdilution wells, rather than produce a diffuse turbidity of the medium, as seen with *Candida* spp. (Figure 3).

In this respect, *Malassezia* is similar to *C. neoformans*, which also tends to precipitate [27]. Accordingly, past experience on this latter organism has shown that agitation of the plates is useful for interpreting end points [71]. Also for *Candida* species, agitation of the plates appears to enhance visual reading and, for both *Cryptococcus* and *Candida*, agitation is deemed necessary before spectrophotometric reading [71]. These findings may suggest that also for *M. pachydermatis* the agitation of the plates prior to reading could make MICs easier to read and more reproducible. However, this lacks experimental evidence, given that this part of the procedure is not detailed in most studies. Our personal experience [34] might even suggest the contrary as we found the size of the button-like deposits of the yeast made the end-point easily quantifiable, while agitation often made this quantitative evaluation more difficult. Moreover, the relative sizes of the buttons correlated well with the OD values obtained by the spectrophotometer. We also noted that in flat-bottomed wells, more uniform suspensions were generated (Figure 1), while deposits of the yeast occurred when U-shape wells were used (Figure 3). Although this technical detail appears to be regarded as of little importance in the literature concerned with AFST of *Malassezia*, with very few studies specifying the type of microplates used, it should be remembered that the form of the wells is an important parameter, given that it is mentioned in the CLSI/EUCAST documents (Table 1) [27].

Two colorimetric methods have been proposed to evaluate MICs for *M. pachydermatis* as an alternative to turbidimetric reading. The first is based on the detection of fungal metabolic activity through an indicator of ox-red activity (Alamar blue). This method was employed to read MICs when tests were performed in a turbid medium, that is the Leeming-Notman broth [43,59]. In the second colorimetric assay, the urea incorporated in the test medium (modified Christensen’s urea broth) was converted to ammonia by the urease activity of the yeast, resulting in an alkaline change in the pH of the medium proportional to the number of surviving yeast, resulting in a visible colour change from yellow/clear to pink using a phenol red pH indicator. The colour change was assessed visually or by a more objective assessment made by a spectrophotometer [50,51,56,58]. Some commercial kits, based on colorimetric (ox-red) reading are available for testing *Candida* spp. The levels of agreement of some of them with the reference methods may support the validity of this indirect approach that is rapid and easy to employ [28]. However, as far as *M. pachydermatis* is concerned, it is logical to think that, since various lipid media that allow an easy assessment of the yeast growth are available, a simpler turbidimetric approach should be standardized, from which more complex indirect colorimetric assays could be developed. Our experience supports this caution, since we employed the urease method and noted that the colour change was often inconstant and sometimes absent (Figure 1). In fact, while urease production is considered as an important trait of *M. pachydermatis* [66], its intensity can vary according to the growth conditions and it can even disappear in strains that have been stored for long periods [38]. Moreover, we suspect that the measurement of colour change instead of the medium turbidity might be too stringent, with consequent underestimation of the antifungal activity. As an example of this effect, the results presented by Nakamura et al. (2000) [51] and Murai et al. (2002) [50] show ITZ MICs several-fold higher than that found by most authors (Table 2).

A third approach for reading MICs in microtitre plates has been proposed. Prado et al. (2008) [57] and Brito et al. (2009) [37] claimed that reading of MICs in microtitre plates was difficult due to the sedimentation of *Malassezia* in the bottom of the wells and proposed overcoming the problem by subculturing aliquots of broth, taken at the end of the incubation period, on potato dextrose agar. Even though MICs obtained by this procedure were similar to those found in most studies, a finding that may demonstrate the validity of this approach, we believe that this modification is cumbersome, time consuming, and unnecessary. We think that MICs for *M. pachydermatis* can be read and interpreted quite easily (see Figure 1 and Figure 3) and that there are other ways to help visual interpretation, such as the use of spectrophotometer reading, which is more suitable. In support of this, a difficult reading of the yeast growth is rarely mentioned in the literature.

Regardless the way the yeast growth is evaluated (turbidimetric vs. colorimetric reading), the MIC endpoint that is adopted is also of major importance [71]. For *Candida*/*Cryptococcus*, CLSI defines the MIC as the lowest drug concentration at which there is substantial reduction of growth (approximately 50%) compared to growth in the drug-free control. Application of this less-stringent endpoint is recommended for azole drugs, due to their fungistatic nature. For *Candida* spp., this phenomenon can result in a partial inhibition of growth over an extended range of antifungal concentrations, with the suspension failing to become optically clear (the so-called trailing end point). Application of this endpoint has improved inter-laboratory agreement and also discriminates between putatively susceptible and resistant isolates of *Candida* spp. [25]. With fungicidal agents such as AMB and TER, growth ceases upon exposure to the drug; this results in clear-cut endpoints. Accordingly, for such agents, the MIC is the lowest drug concentration that results in optically clear medium [72].

It is hard to know now which end point determination will produce the most reproducible and representative MICs for *M. pachydermatis*. Concerning the azoles, some authors have referred to an MIC definition as the level of 50% inhibition of growth compared with drug-free growth control (which mirrors the MIC endpoint recommended for *Candida* and *Cryptococcus*), whilst others used a 90%, 99%, or 100% inhibition level. Velegraki et al. (2004) [62] found that the trailing growth phenomenon was only weakly displayed by *Malassezia* spp. and they therefore suggested a 90% endpoint. However, their study was mainly focused on other *Malassezia* species and included only one strain of *M. pachydermatis*. It is also unclear if different end points should be applied to different antifungals, as for *Candida* testing. It appears that some authors have maintained the same MIC endpoint for azoles and AMB [38] and for azoles and TER [50], while others have adapted it according to the antifungal class [37,57,62].

#### 3.1.7. The Quality Controls (QC)

The goals of a QC program are the following: (i) the precision (and repeatability) and accuracy of the susceptibility test procedure; (ii) the performance of the reagents, testing conditions, and instructions used in the test; and (iii) the performance of persons who conduct the tests and read the results [25]. The goals are well realized by, although not limited to, the use of reference strains. By including reference strains that are known to provide defined MICs/inhibition diameters under the test conditions employed, the user can monitor the performances of the laboratory procedure over time. Moreover, it is useful to compare inter-laboratory results. CLSI standards include recommendations for specified strains from the American Type Culture Collection (ATCC) to be used as QC strains [25,26,29]. Due to the lack of a standard testing methodology, QC strains of *M. pachydermatis* are not available yet. As reported above, some authors overcome this problem by using the *Candida* and *Cryptococcus* QC strains [57,58]. In other cases, random repetitions of the tests were performed to test the reproducibility of the results obtained, while some authors did not monitor their results by any QC measure.

## 4. Conclusions and Perspectives

Analogous to the standardization of *Candida*/*Cryptococcus* susceptibility testing, it is likely that the analysis and standardization of each of the test condition parameters might increase the reproducibility of methods for susceptibility testing of *M. pachydermatis*. It is advisable to develop a standard procedure that can be carried out through a multicenter approach, resulting in broad consensus from the scientific community. Some of the testing options reported in the literature appear suitable and worth adopting in a future reference standard dedicated to this yeast. A microtitre plate broth format, with turbidity as a growth indicator and a medium as simple and definable as possible, with any lipid supplementation which is often cited as the cause of drug interference [73] reduced to the minimum that is necessary to support the yeast growth, is likely to represent the best starting point. Avoiding the unnecessary use of lipid supplementation will also reduce the difficulty encountered in interpreting endpoints due to the added turbidity afforded by the lipid enrichment. The two culture media that may be now considered the most suitable for testing *M. pachydermatis* are Christensen’s broth with Tween 40/80 and Sabouraud Broth with Tween 80, because they were shown to not antagonize the drug activity and to support the adequate growth of the yeast [15,33,34,40,58]. Moreover, they have a well-defined and reproducible formula.

Other test formats may be of interest as well, e.g., agar dilution procedures. For this type of assay, Leeming–Notman agar may be recommended since it was shown not to antagonize the activity of the drugs under testing [45]. The E-test^®^ method also appears promising, as it allows MIC values to be obtained, but via a more simplified agar diffusion procedure. However, a limitation is that the strips are not commercially available for all antifungal agents used in dogs.

On the other hand, we think that the disk diffusion technique, although more practical and easy to apply, should be employed only once a reproducible broth-based technique is available. In fact, this will represent the basis of verifying the functioning of disk-diffusion and the correlation of inhibition zones with MIC values. For the disk diffusion format, we would suggest the use of Mueller-Hinton Agar with glucose and methylene blue, for the reasons discussed above.

For all formats, much work must still be done to standardize the inoculum preparation. The use of dispersing agents, such as Tween, together with mechanical methods, will probably guarantee the best result in dispersing *Malassezia* clumps, thus facilitating the production of homogenous suspensions with reproducible CFU. Following this, parameters will have to been determined as far as the spectrophotometer reading is concerned (i.e., the wavelength, the OD values, and the corresponding number of CFU).

Regarding the MIC definition in the broth and agar dilution assays, further experiments are necessary with a large number of isolates to clarify the “in vitro behaviour” of *M. pachydermatis* in the presence of each of the antifungal drug classes that possess different mechanisms of action, and the consequent optimal end point for each of them.

For any procedure, another key step will be the establishment of a QC regimen with specified strains of *M. pachydermatis* selected as QC strains. For this selection, it should be taken in account that the ideal reference strains for QC of the dilution methods have MICs that fall near the midrange of the concentration for all of the antifungal agents tested. Moreover, before a strain is accepted as a reference, it should be tested for as long as is necessary to demonstrate that its antifungal susceptibility pattern is genetically stable [25]. One candidate could be the already cited CBS strain 1879 which will be sustainable and readily accessible as it is already stored in a culture collection. The use of reference strains will also allow the comparison of results between different laboratories and published reports.

In conclusion, once a method has been shown to provide results with good inter- and intra-laboratory agreement, the challenge will be to evaluate the correlation of in vitro data with clinical outcomes. This will be mandatory in order to ascertain the clinical relevance of these in vitro determinations.

## Figures and Tables

**Table 1:** Main parameters for the performance of the Clinical and Laboratory Standards Institute (CLSI) broth methods (M27-A3 and M27-S4 documents) (with European Committee on Antibiotic Susceptibility Testing (EUCAST) modifications) and disk diffusion method (M44-A and M44-S2 documents) for yeasts. Data from Arikan (2007) [28] and Canton et al. (2009) [27].

Parameter	Broth-Method	Disk-Diffusion Method
Test medium	Roswell Park Memorial Institute Medium (RPMI-1640) with glutamine, without bicarbonate. Glucose concentration: 0.2% (EUCAST 2%)	Mueller-Hinton agar + 2% glucose + 0.5 mg/L methylene blue
Inoculum size	0.5 × 10^3^–2.5 × 10^3^ CFU/mL (EUCAST 1 × 10^5^–5 × 10^5^)	0.5 Mc Farland standard (1 × 10^6^ to 5 × 10^6^ CFU/mL)
Microdilution plates	96 U-shaped wells (EUCAST flat-bottom wells)	NA
Temperature and incubation time	*Candida* spp., 48 h ^a^ at 35 °C (EUCAST 24 h) *Cryptococcus neoformans*, 72 h at 35 °C	35 °C for 20–24 h Some strains of *Candida glabrata*, *Candida krusei*, and *Candida parapsilosis* often require 48 h
Reading method	Visual (EUCAST Spectrophotometric 530 nm)	Measurement of zone size

^a^ = Reading at 24 h is acceptable, provided that fungal growth is adequate, for amphotericin B, and fluconazole. Echinocandins must be read at 24 h; NA = not applicable.

**Table 2:** Some minimum inhibitory concentrations (MICs, µg/mL) of different antifungal agents against isolates of *M. pachydermatis* reported in the literature.

Drug	Ref.	Format	N°	Range	MIC_50_	MIC_90_	CBS 1879
AMB	Prado et al. (2008) [57]	BMD	50	ND	ND	2 ^a^	Not tested
	Brito et al. (2009) [37]	BMD	20	0.25 ^b^	0.25	0.25	Not tested
	Velegraki et al. (2004) [62]	BMD	1	0.12 ^b^	NA	NA	Not tested
		E-test	1	0.5 ^b^	NA	NA	Not tested
	Brito et al. (2007) [38]	AD	32	0.125–8	0.5	8	Not tested
MCZ	Uchida et al. (1990) [61]	BMD	42	0.16–>80	1.25	20	2.5
	Gordon et al. (1988) [44]	BMD	7	0.009–0.039	ND	ND	Not tested
	Pietschmann et al. (2008) [56]	BMD	1	2.92	NA	NA	2.92
	Hensel et al. (2009) [46]	BMD	24	0.03–0.5	0.125	0.25	Not tested
	Peano et al. (2012) [34]	BMD	51	0.03–16	2	4	Not tested
ITZ	Murai et al. (2002) [50]	BMD	24	1.6 ^b^	1.6	1.6	1.6
	Garau et al. (2003) [43]	BMD	10	≤0.03–0.06	≤0.03	0.06	Not tested
	Eichenberg et al. (2003) [42]	BMD	82	0.007–0.125	0.06	0.125	Not tested
	Rincon et al. (2006) [58]	BMD	3	0.03–0.125	NA	NA	0.06
	Prado et al. (2008) [57]	BMD	50	ND	ND	<0.03 ^a^	Not tested
	Brito et al. (2009) [37]	BMD	20	≤0.03–0.25	≤0.03	≤0.03	Not tested
	Jesus et al. (2011) [17]	BMD	30	0.01–1	0.125	0.5	Not tested
	Nascente et al. (2003) [19]	BMD	24	0.03–4	0.125	0.5	Not tested
		E-test	35	0.002–2	0.003	0.016	Not tested
	Velegraki et al. (2004) [62]	BMD	1	0.06 ^b^	NA	NA	Not tested
		E-test	1	0.12 ^b^	NA	NA	Not tested
	Nijma et al. (2011) [54]	BMD	30	<0.03–2	<0.03	<0.03	<0.03
		E-test	30	<0.03–8	<0.03	<0.03	<0.03
	Nakamura et al. (2000) [51]	BMD	12	0.8–6.3	ND	ND	Not tested
		AD	12	0.4–6.3	ND	ND	Not tested
	Gupta et al. (2000) [45]	AD	4	≤0.03 ^b^	NA	NA	≤0.03
	Sugita et al. (2005) [60]	AD	6	0.016 ^b^	NA	NA	Not tested
	Brito et al. (2007) [38]	AD	32	≤0.0075 ^b^	≤0.0075	≤0.0075	Not tested
TER	Murai et al. (2002) [50]	BMD	24	3.2 ^b^	3.2	3.2	3.2
	Weseler et al. (2002) [65]	BMD	9	0.2–1.6	NA	NA	Not tested
	Velegraki et al. (2004) [62]	BMD	1	0.12	NA	NA	Not tested
		E-test	1	NA ^c^	-	-	Not tested
	Gupta et al. (2000) [45]	AD	4	≤0.03 ^b^	NA	NA	≤0.03

AMB = Amphotericin B; MCZ = miconazole; ITZ = itraconazole; TER = terbinafine; BMD = microdilution broth format; AD = agar dilution; N° = number of isolates tested; ND = not defined; NA = not applicable since <10 strains tested; ^a^ = the MIC was defined as the lowest antifungal concentration with the lowest number of strains with colony counting similar to the drug-free control for azole drugs, or the lowest concentration that completely inhibited fungal growth for AMB; ^b^ = no range, only one strain tested or MICs equal for all of the strains; ^c^ = E-Test strip was not available for TER. The last column documents the MICs obtained for the reference strain of *M. pachydermatis* CBS 1879 (collection of the Westerdijk Fungal Biodiversity Institute, formerly the Fungal Biodiversity Center—CBS, Utrecht, The Netherlands).

## Supplementary Materials

The following are available online at www.mdpi.com/2309-608X/3/3/37/s1, Table S1: Detailed description of previous studies concerned with in vitro susceptibility testing of *M. pachydermatis* (test format, test conditions, main results, etc.).
